# Supplementary material for: Inducible cell-to-cell signaling for tunable dynamics in microbial communities
Source: Nat Commun. 2020 Mar 4;11:1193. doi: 10.1038/s41467-020-15056-8 (PMC7055273; doi:10.1038/s41467-020-15056-8)
Supplement: Supplementary file 1 — Supplementary Information [file 41467_2020_15056_MOESM1_ESM.pdf]

Supplementary Information

Inducible cell-to-cell signaling for tunable

dynamics in microbial communities

Miano et al.

**Strain name**	**Strain #**	**Host Strain**	**Plasmids**	**Referenced in Figure**
AM15	iQS	MG1655	pTD103-RpaR-sfGFP + pAM21	Fig. 2c, Fig. 2d
AM09	LEAKY	MG1655	pTD103-RpaR-sfGFP + pZA35 X714E (+RpaR)	Ext. data 2
AM010	iSLC	MG1655	pTD103-RpaR-RpaI-LAA-sfGFP + pAm014	Ext. data 1, Fig 2e, Fig 2f, Fig. 3b, Fig. 3c, Fig. 3d, Fig 4.b, Fig 4.c, Fig 4.b, Ext. data 4, Ext. data 6
AM013	SLC	MG1655	pTD103-LuxR-LuxR-LAA-CFP + pZA35 X714E (+LuxR)	Fig 4.b, Fig 4.c, Ext. data 5, Ext. data 6

Supplementary Table 1| The strains used in this study.

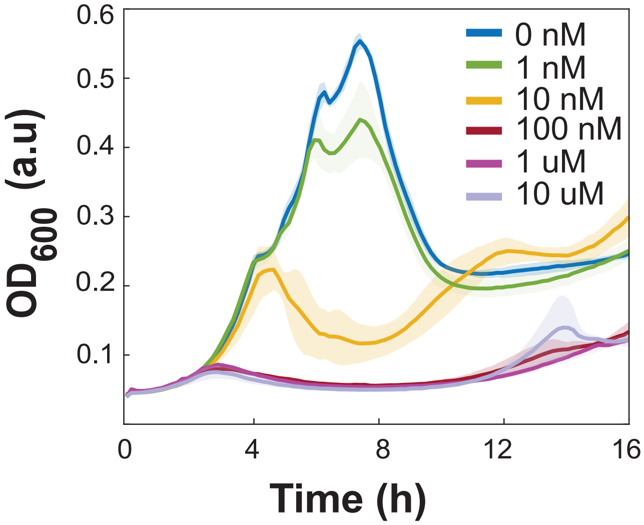

Supplementary Figure 1| iSLC growth curves from plate reader experiments obtained with varying concentrations of p-coumaric acid. Lines and shaded areas represent the mean and standard deviation (n = 3) respectively.

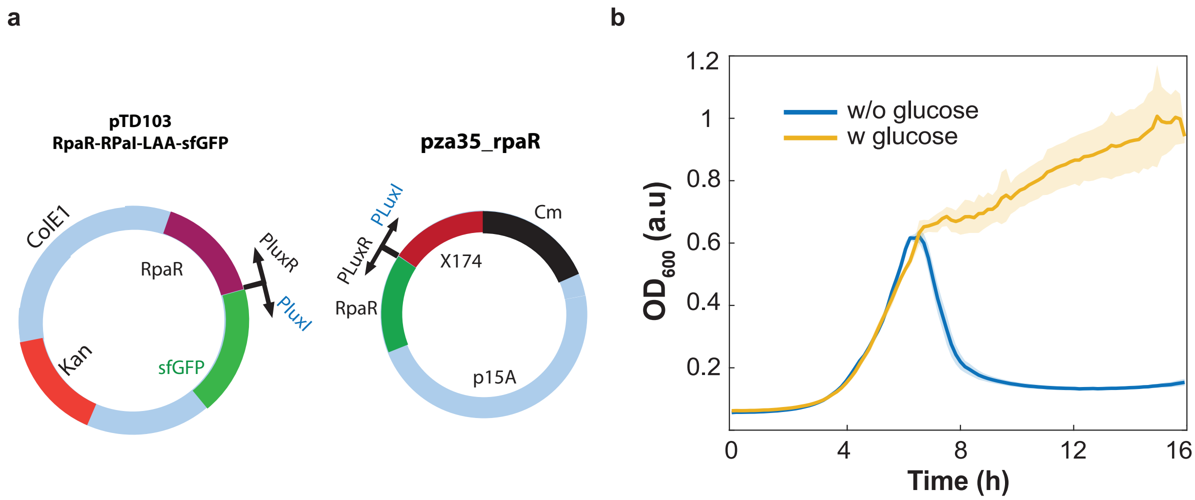

Supplementary Figure 2| **a,** Plasmids maps from strain LEAKY which lacks the Rpa cassette (RpaI - Plux - RpaR). **b,** Plate reader data obtained by growing strain LEAKY in presence and absence of glucose respectively. High glucose levels in the cell correspond to low cAMP levels which is involved into the luxI promoter activation due to the presence of a binding site for the CAP-cAMP activating complex.[1]

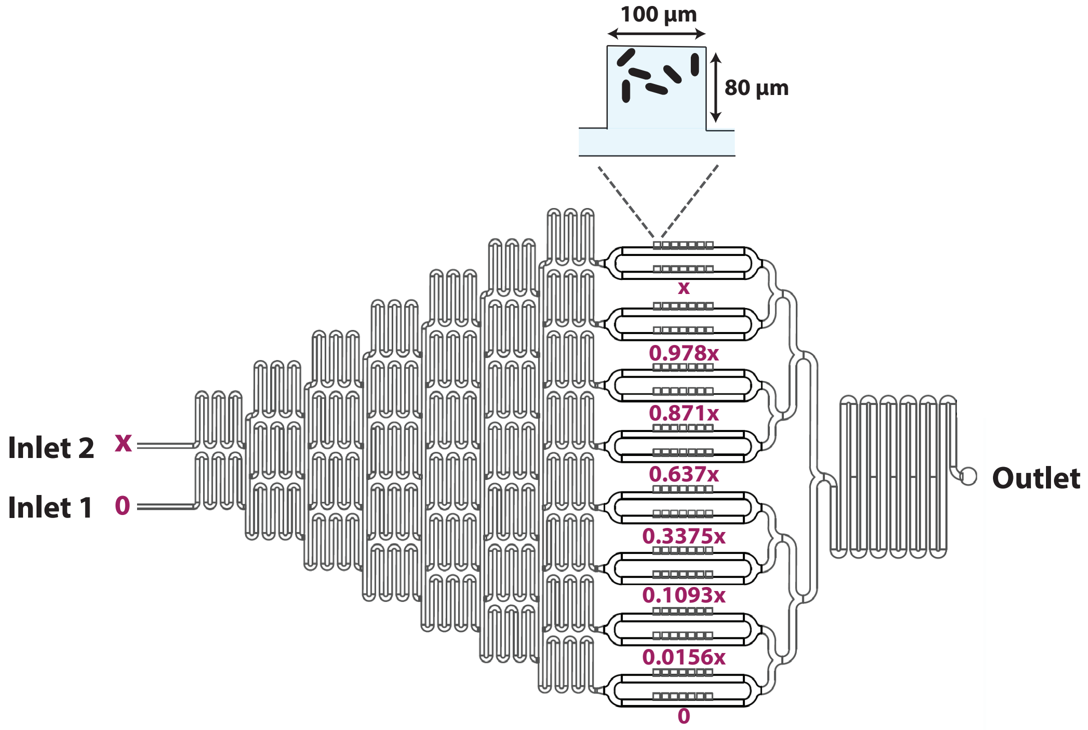

Supplementary Figure 3| Microfluidic gradient device used for experiments in Fig. 2 and Fig. 4. Inlet 1 is set to zero inducer concentration, while Inlet 2 is set to concentration X to generalize dilution factors.

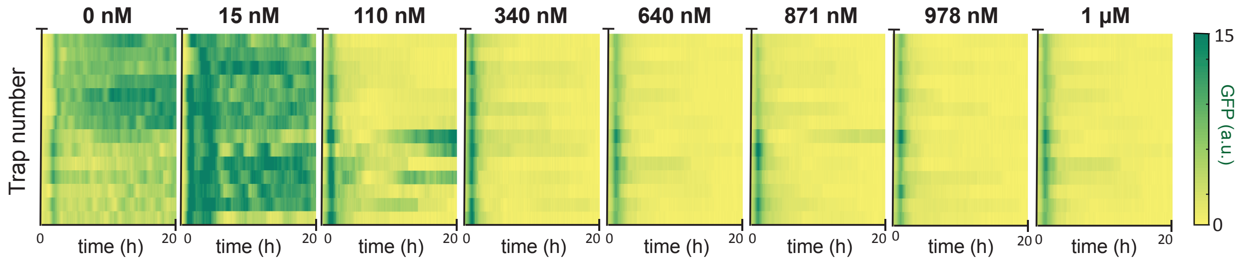

Supplementary Figure 4| Complete heatmaps of fluorescence data for experiment described in Fig. 2. The approximate p-coumaric acid concentration present in each column is reported at the top. For each column, all fourteen traps are reported.

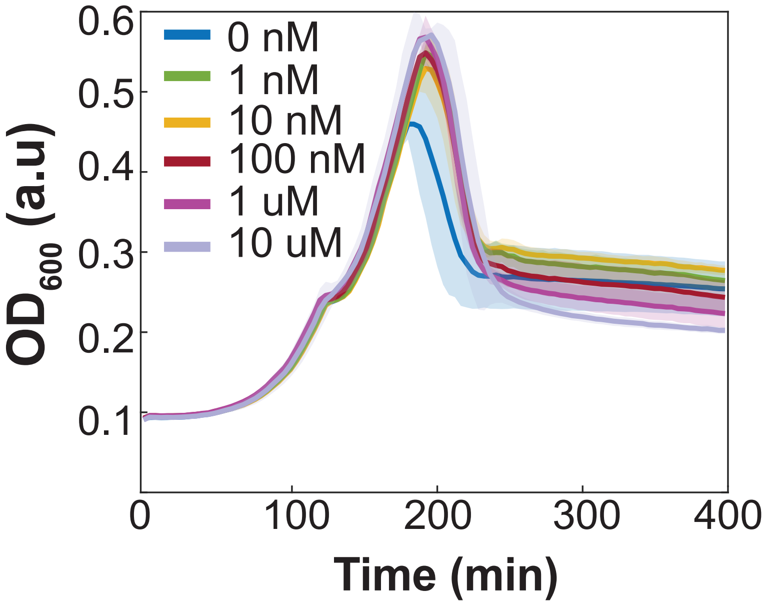

Supplementary Figure 5| Growth curves from plate reader experiments for the iSLC strain. For all p-coumaric acid concentrations tested the growth curves are unaffected, confirming complete orthogonality to the compound. Lines and shaded areas represent the mean and s.d. (n = 3) respectively.

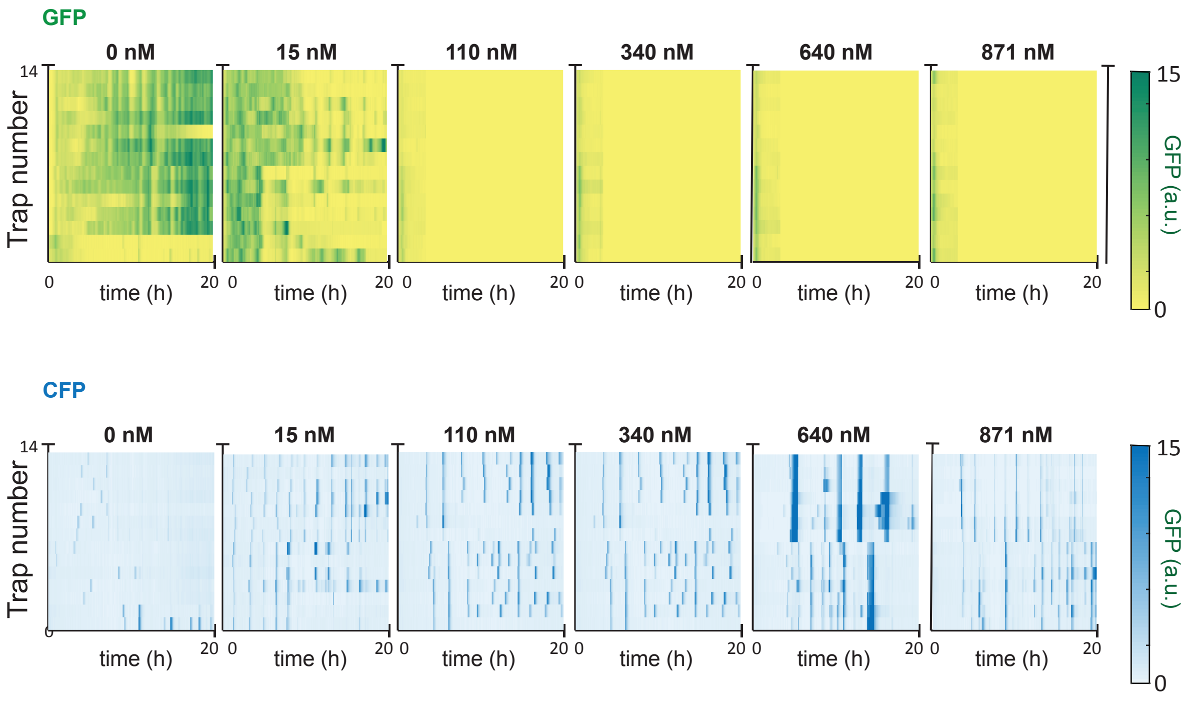

Supplementary Figure 6| Complete heatmaps of fluorescence data for the experiment described in Fig. 4. The approximate p-coumaric acid concentration present in each column is reported at the top. For each column, all fourteen traps are reported. GFP is reported in the top row. CFP is reported in the bottom row.

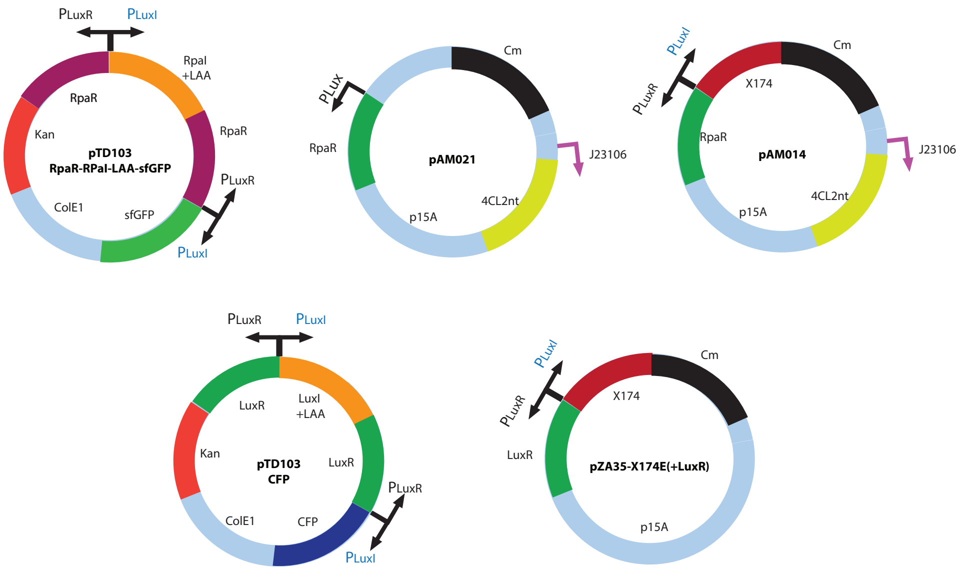

Supplementary Figure 7: Plasmids used in this study. LAA stands for the LAA ClpXP mediated degradation tag.

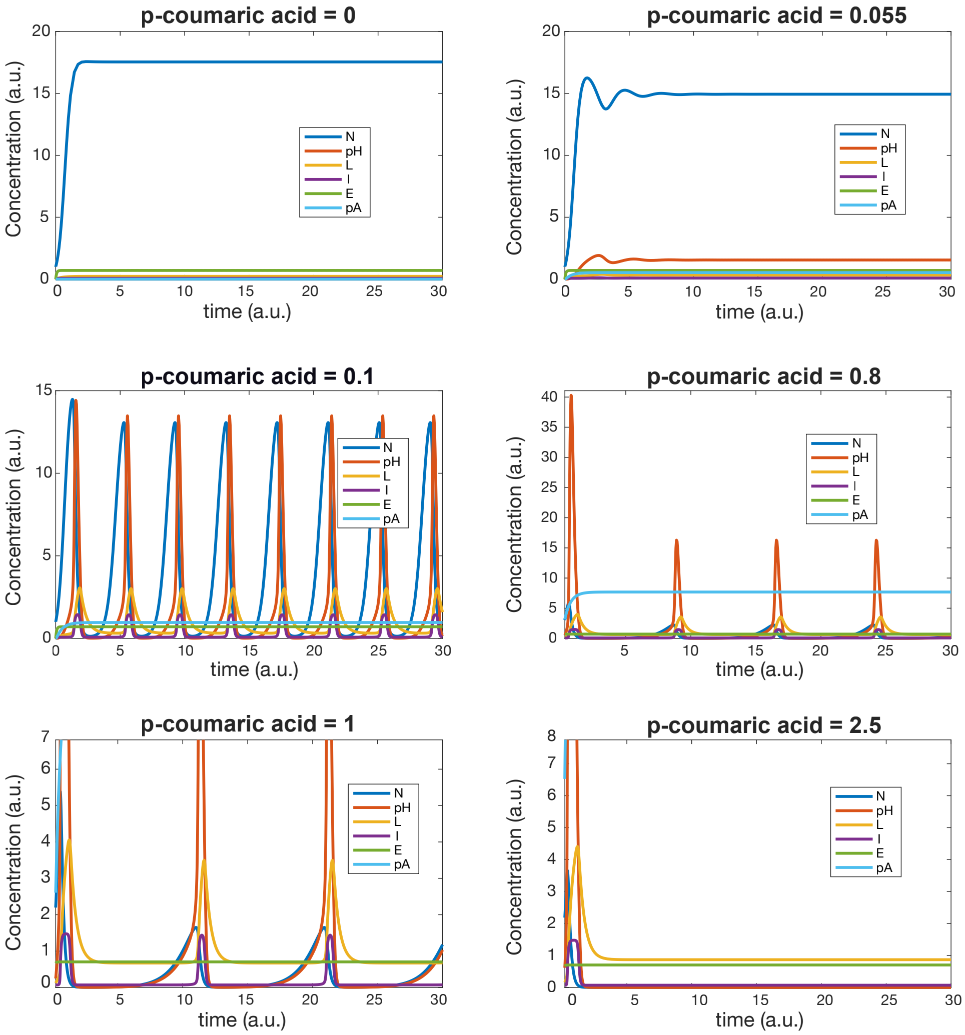

Supplementary Figure 8: Simulation of the evolution of all six variables over time. Six p-coumaric acid concentrations are considered to span the emerging population dynamics. Initally, all the variables are set to zero except cell number (N) which is set to 1.

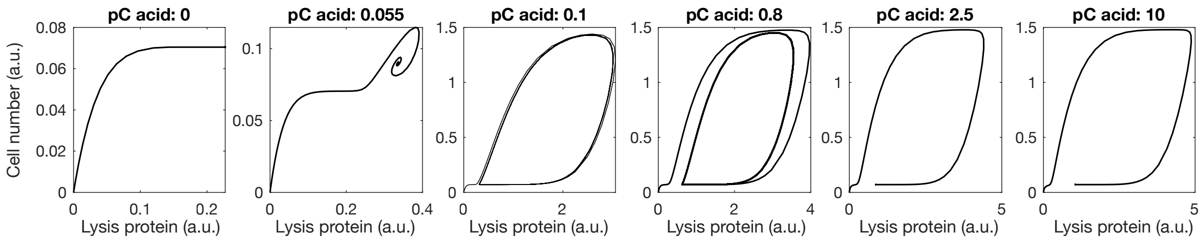

Supplementary Figure 9: Phase portraits of variables N (cell number) and L (lysis protein) showing a Hopf bifurcation caused by the appearance and disappearance of a limit cycle.

References

[1] Meighen, E. A. Genetics of bacterial bioluminescence. *Annual review of genetics* **28**, 117–139 (1994).
