# Supplementary material for: Inducible cell-to-cell signaling for tunable dynamics in microbial communities
Source: Nat Commun. 2020 Mar 4;11:1193. doi: 10.1038/s41467-020-15056-8 (PMC7055273; doi:10.1038/s41467-020-15056-8)
Supplement: Supplementary file 3 — Description of Additional Supplementary Files [file 41467_2020_15056_MOESM3_ESM.pdf]

Description of Additional Supplementary Files

File Name: Supplementary Movie 1
Description: iSLC strain subject to multiple concentrations of pcoumaric acid in the gradient microfluidic device with traps of dimensions 100 x 80 x 1.2 µm. Images taken at 10x magnification. Multiple inducer concentrations results in different population dynamics.

File Name: Supplementary Movie 2
Description: SLC strain subject to three concentrations of pcoumaric acid in the gradient microfluidic device with traps of dimensions 100 x 80 x 1.2 µm. Images taken at 60x magnification. Zero p-coumaric acid results in circuit quiescence with low level expression due to promoter leakiness. Intermediate inducer concentration (15 nM) corresponds to circuit activation with sustained oscillations in population density. High concentration (110 nM) results in total strain killing with no survivors left.

File Name: Supplementary Movie 3
Description: iSLC strain kill switch response to 500 nM pcoumaric induction at time zero in microfluidic device with traps of dimensions 100 x 80 x 1.2 µm. Images taken at 4x magnification. Number of culture regions in the chip is 406.

File Name: Supplementary Movie 4
Description: Co-culture of iSLC (green) and SLC (blue) strain (seeded at 1:1 ratio) subject to multiple concentrations of p-coumaric acid in the gradient microfluidic device with traps of dimensions 100 x 80 x 1.2 µm. Images taken at 10x magnification. Different inducer concentrations result in three emerging multi-population dynamics.
